# Prostatic Stromal Hyperplasia with Atypia

**DOI:** 10.1155/2013/364124

**Published:** 2013-05-28

**Authors:** Ryan C. Hutchinson, Kevin J. Wu, John C. Cheville, David D. Thiel

**Affiliations:** ^1^Department of Urology, Mayo Clinic, 4500 San Pablo Road, Jacksonville, FL 32224, USA; ^2^Department of Pathology, Mayo Clinic, 4500 San Pablo Road, Jacksonville, FL 32224, USA; ^3^Department of Pathology, Mayo Clinic, 200 1St Street SW, Rochester, MN 55905, USA

## Abstract

Prostatic stromal hyperplasia with atypia (PSHA) is a rare histologic finding diagnosed incidentally on prostate biopsies, transurethral resection specimens, and radical prostatectomy specimens. PSHA has a bizarre histologic appearance and these lesions often raise concern for sarcoma; however, their clinical course is indolent and does not include extraprostatic progression. We discuss a case of PHSA discovered on prostate biopsy performed for an abnormal digital rectal examination and review the literature on this rare pathologic finding.

## 1. Introduction

Prostatic stromal hyperplasia with atypia (PSHA) is a rare histologic finding diagnosed incidentally in specimens from transrectal ultrasound (TRUS-) guided needle biopsy of the prostate, transurethral resection of prostate (TURP), radical prostatectomy, and simple prostatectomy [1]. Because of their bizarre histologic appearance, these lesions raise concern for sarcoma; however, their clinical course is indolent and does not include extraprostatic progression. 

## 2. Case Presentation

**Figure 1:**
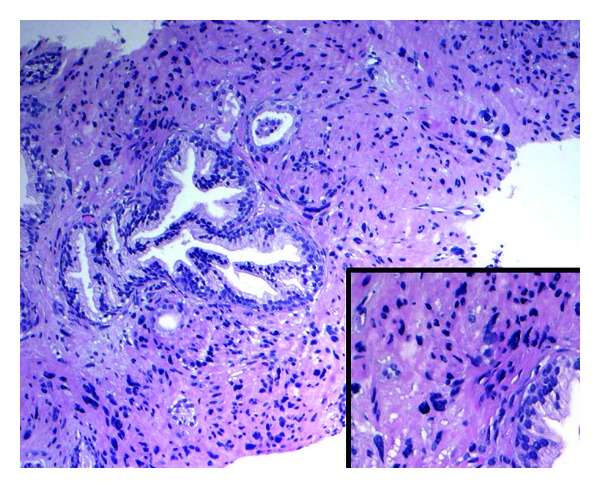
H/E stain, low-power magnification (10x) with high-power inset (20x) prostatic tissue showing hypercellular stroma with atypical hyperchromatic nuclei surrounding benign prostatic glands present focally in one of ten needle biopsies. Inset shows a higher power view of the atypical stromal cells with smudgy chromatin and adjacent normal prostatic acinus in lower left.

A 55-year-old man underwent a 10-core TRUS biopsy for a grossly abnormal digital rectal exam. Histologic examination (Figure 1) revealed hypercellular stroma with hyperchromatic nuclei around benign prostatic glands in 1 of the 10 cores. There was an absence of adenocarcinoma in the remaining cores. High-power examination revealed smudgy chromatin within these cells (Figure 1 inset). The patient was reassured and placed on watchful waiting with yearly PSA examinations.

## 3. Discussion

 PSHA is characterized by one or more ill-defined, uncircumscribed, and hyperplastic stromal nodules infiltrating around benign acini [2]. Immunohistochemical staining further confirms the diagnosis by demonstrating intense immunoreactivity for androgen receptors, while being devoid of activity for estrogen receptors or Ki-67. In contrast with prostatic leiomyoma with atypia, these cells are intensely immunoreactive for vimentin instead of desmin and actin [3]. 

PSHA does not generally present as a symptomatic lesion in and of itself, though symptomatic cases have been reported [4]. In all cases reported, the portion of tissue comprised of PSHA was between 5–20% of the tissue, with the rest generally being typical nodular hyperplasia [1]. These lesions, despite their atypical appearance, have a universally benign course and no case of metastatic disease has been reported, though some patients undergoing surgical management for BPH have required re-resection [5].

 This finding has been referred to by a variety of names including: atypical stromal hyperplasia, symplastic leiomyoma, and pseudoneoplastic lesion of the prostate gland. PSHA was previously grouped with low malignant potential findings such as phyllodes tumor and low-grade sarcoma as stromal tumors of unknown malignant potential (STUMP); however, given the univerally benign course of PSHA, this may constitute a misnomer. The current nomenclature emphasizes the expected indolent clinical course with treatment focused on the original disease of interest [3].
